# In Vitro Degradation of 3D-Printed Poly(L-lactide-Co-Glycolic Acid) Scaffolds for Tissue Engineering Applications

**DOI:** 10.3390/polym15183714

**Published:** 2023-09-09

**Authors:** Anushree Ghosh Dastidar, Susan A Clarke, Eneko Larrañeta, Fraser Buchanan, Krishna Manda

**Affiliations:** 1 School of Mechanical and Aerospace Engineering, Queen’s University Belfast, Belfast BT9 5AH, UK; aghoshdastidar01@qub.ac.uk (A.G.D.); f.buchanan@qub.ac.uk (F.B.); 2 School of Nursing and Midwifery, Queen’s University Belfast, Belfast BT9 7BL, UK; s.a.clarke@qub.ac.uk; 3 School of Pharmacy, Queen’s University Belfast, Belfast BT9 7BL, UK; e.larraneta@qub.ac.uk

**Keywords:** poly(L-lactide-co-glycolide), accelerated, degradation, cartilage tissue engineering, bioresorbable

## Abstract

The creation of scaffolds for cartilage tissue engineering has faced significant challenges in developing constructs that can provide sufficient biomechanical support and offer suitable degradation characteristics. Ideally, such tissue-engineering techniques necessitate the fabrication of scaffolds that mirror the mechanical characteristics of the articular cartilage while degrading safely without damaging the regenerating tissues. The aim of this study was to create porous, biomechanically comparable 3D-printed scaffolds made from Poly(L-lactide-co-glycolide) 85:15 and to assess their degradation at physiological conditions 37 °C in pH 7.4 phosphate-buffered saline (PBS) for up to 56 days. Furthermore, the effect of scaffold degradation on the cell viability and proliferation of human bone marrow mesenchymal stem cells (HBMSC) was evaluated in vitro. To assess the long-term degradation of the scaffolds, accelerated degradation tests were performed at an elevated temperature of 47 °C for 28 days. The results show that the fabricated scaffolds were porous with an interconnected architecture and had comparable biomechanical properties to native cartilage. The degradative changes indicated stable degradation at physiological conditions with no significant effect on the properties of the scaffold and biocompatibility of the scaffold to HBMSC. Furthermore, the accelerated degradation tests showed consistent degradation of the scaffolds even in the long term without the notable release of acidic byproducts. It is hoped that the fabrication and degradation characteristics of this scaffold will, in the future, translate into a potential medical device for cartilage tissue regeneration.

## 1. Introduction

The aim of cartilage tissue engineering is to regenerate and/or enhance the functionality of damaged articular cartilage by utilising degradable matrices called scaffolds to assist in cell adhesion, proliferation, and growth to produce viable tissue [1]. The scaffolds’ structure and surface morphology must satisfy several criteria specific to the targeted tissue for it to function as a temporary matrix: (i) a three-dimensional porous architecture to ensure cell growth, nutrient flow, and metabolic waste transport; (ii) comparable mechanical properties; (iii) appropriate surface chemistry, and (iv) controllable degradation [2].

The degradation rate of the polymer is a critical characteristic as it will affect cellular interactions with the scaffold, including cell proliferation, tissue regeneration, and host tissue response [3]. The scaffold’s degradation performance must match the rate at which the cartilage regenerates to provide sufficient biomechanical support to allow for simultaneous tissue development and replacement [4]. Based on the clinical application, the bioresorbable polymer would ideally sustain adequate mechanical properties for about 8–12 weeks [5,6,7]. Members of the poly (α-hydroxy acid) family, such as Polyglycolide (PGA), Polylactide (PLA), and their related copolymers like Polylactic-co-glycolic acid (PLGA) have been employed in the past [8] with PLA found in the L enantiomer form, namely poly(L-lactide) (PLLA), a popular choice for biomedical applications. Since the use of copolymers enables greater control of mechanical properties, as well as degradation rates, the copolymerisation of PLLA and PGA, forming Poly(L-lactide-co-glycolide) (PLLGA) is an excellent material for load-bearing applications due to its superior mechanical strength and semi-crystalline structure [9]. 

The different variables that can affect the degradation rate of PLGA are (i) molecular weight and (ii) the ratio of Lactic acid to Glycolic acid [10]. Due to its reasonable degradation (~6–12 months) and comparable biomechanical properties (compressive modulus 1–2 MPa) to the native cartilage (compressive modulus 0.45–0.8 MPa), PLGA with high Lactic acid-to-Glycolic acid ratio and ester end cap can make an effective scaffold for cartilage regeneration [11,12]. It is for this reason that PLLGA 85:15 has been selected as the material for this study that has not been widely evaluated in the past as a scaffold for cartilage tissue engineering applications. The utilisation of a high Lactic acid-to-Glycolic acid ratio like PLLGA 85:15 is hypothesised to provide stable degradation and to support biocompatibility in the host.

PLGA scaffolds to date have mostly been fabricated using manufacturing methods that allow limited control over the architecture and porosity of the scaffolds, which can be a concern for reproducibility, mechanical properties, and cartilage regeneration [9,10,13,14]. A scaffold with a porous, interconnected structure is a prerequisite for tissue regeneration because it enables the efficient mass transfer of oxygen and nutrients for infiltrating cells and a suitable surface area for cell proliferation [15,16]. 3D printing offers previously unheard-of versatility, with exact control over pore characteristics [17]. Studies in the past have examined the in vitro degradation of PLGA scaffolds; however, none used porous 3D-printed scaffolds that have biomechanical properties comparable with the native cartilage [3,9,18,19,20].

Hence, this study aims to develop porous 3D-printed PLLGA 85:15 scaffolds that have biomechanical properties comparable with native cartilage.

The degradation of these scaffolds will be assessed under physiologically simulated in vitro degradation conditions, and initial in vitro cell viability studies will assess the effect of degradative changes in the scaffolds on cell attachment and proliferation. Evaluating the long-term degradation characteristics of polymers could take months or years to decompose at physiological temperatures. The ability to extrapolate accelerated test results back to physiological conditions via temperature-accelerated degradation has shown potential in assessing the long-term degradation of scaffolds [21,22,23,24]. Hence, an experimental evaluation of accelerated degradation has also been conducted to understand the long-term behaviour of the scaffolds and compare the rate of degradation to physiological degradation in these scaffolds.

## 2. Materials and Methods

Pelletised PLLGA 85:15 (Purac, PURASORB PLG 8531, Corbion Biomaterials, Amsterdam, The Netherlands) were utilised for this study. Before use, materials were kept in sealed packing at −20 °C. PLLGA pellets were dried in a vacuum oven for 4 h at 80 °C, 75 mm Hg. Scaffolds were printed using a GeSiM BioScaffolder 3.1 (GeSiM mbH, Großerkmannsdorf, Germany) with 300 μm nozzle, at 190 °C with an extrusion speed of 5.5 mm s^−1^, filament spacing of 1.7 mm. The scaffolds were of 8 × 8 mm size by depositing an orthogonal array of 8 layers with fibre direction changed by 90° after every two layers (∼3 mm total construct height).

### 2.1. Characterisation of the Fabricated Scaffolds—Porosity, Pore, and Strut Sizes

Optical microscopy was performed on the scaffold to image the scaffold structure and cross-section using a Nikon SMZ800 Zoom Stereomicroscope (Nikon Instruments, Tokyo, Japan). Image J v1.51q (National Institute of Mental Health, Bethesda, MD, USA) was used to measure pore and strut size. A Scanning Electron Microscope (SEM) JEOL 6500 FEG SEM (Advanced Microbeam Inc., Vienna Center, OH, USA) was used to image scaffolds for closer inspection of pore and strut formation and to identify any printing errors. Scaffolds were coated with 10–20 nm of gold using an Auto Sputter Coater (Agar Scientific, Stansted, UK). To examine internal side geometry, scaffolds were also sectioned along the Z plane using a scalpel to expose the cross-sectional area. The initial porosity of the scaffold was measured using the liquid displacement method [25], where each scaffold (n = 4) was submerged in a known volume of ethanol (V_1_). The final volume of the liquid and the liquid-impregnated scaffold was measured as V_2_, and the volume of the remaining liquid when the liquid-impregnated scaffold was removed as V_3_. The porosity of the scaffold was then calculated using Equation (1).

(1) $$\mathrm{Porosity}=\frac{V_{1}-V_{3}}{V_{2}-V_{3}}$$

### 2.2. In Vitro Simulated Physiological Condition Degradation

After rinsing with sterile PBS (pH 7.4) and washing in ethanol under sterile conditions in a Class II biosafety hood, the scaffolds were degraded in vitro according to ISO 13781:2017 [26] for 56 days by immersing in 10 mL PBS with a volume-to-mass ratio of approximately 140:1. The scaffolds were maintained at a physiologically relevant temperature of 37 °C. Three scaffolds at each time point (3, 7, 10, 14, 21, 28, 42, and 56 days) were retrieved for characterisation, and the PBS solution remained unchanged for the entire duration of the degradation tests. 

#### 2.2.1. Swelling, Mass Loss, pH

The scaffolds’ original mass, m_0_, was measured, and at each time point, the degraded scaffolds were rinsed thoroughly with distilled water before being dried with Kimtech science precision wipes to eliminate surface moisture and weighed to calculate the wet mass (m_w_). To determine the dry mass of the scaffolds (m_d_), they were dried in a vacuum oven at 30 °C for 8 h at 600 mm Hg and weighed. At each time point, the percentage swelling and the percentage mass loss were calculated using Equations (2) and (3). The pH of the PBS solution was recorded at each time point to investigate the change of acidity over time.

(2) $$\%\mathrm{Swelling}=\frac{m_{w}-m_{d}}{m_{d}}\times 100\%$$

(3) $$\%\mathrm{Mass}\;\mathrm{loss}=\frac{m_{0}-m_{d}}{m_{0}}\times 100\%$$

#### 2.2.2. Thermal Properties and Crystallinity

The change in the thermal characteristics of the degrading scaffolds was determined using Differential Scanning Calorimetry (DSC) (Perkin Elmer DSC6, Perkin-Elmer, Waltham, MA, USA) to determine the glass transition temperature (T_g_) and any indications of crystallisation. Samples (8–10 mg) were sealed in an aluminium pan and scanned in a nitrogen atmosphere. A thermal cycle was conducted from 20 °C to 200 °C at a rate of 10 °C/min in a two-stage heating process. The second heating cycle of PLLGA has been employed for further analysis. Utilizing TA instruments’ Universal Analysis software 2000, measurements of the T_g_, melting temperature (T_m_), and enthalpy of fusion (H_melt_ J/g) were made. The T_g_ was established as the midpoint of the transition, while the T_m_ was identified as the peak temperature. When two peaks were seen, the highest peak was designated as the T_m_. Since a H_melt_ for a 100% crystalline sample of PLLGA is not available, the percent crystallinity of PLLGA was calculated using Equation (4) utilising the H_melt_ values obtained for PLLGA relative to the H_melt_ of a 100% crystalline sample of PLLA, H_melt_ = 93 J/g [22].

(4) $$\%\mathrm{Crystallinity}=\frac{\Delta H_{\mathrm{melt}\;}\left(J/g\right)}{93\;\left(J/g\right)}\times 100\%$$

#### 2.2.3. Molecular Weight Changes

Gel permeation chromatography (GPC) experiments were performed to determine variations in the molecular weight during scaffold degradation. This was conducted using an Agilent 1260 Infinity II GPC with two PLgel 5 μm MIXED-C columns (PS/DVB), a PLgel 5 μm guard column running at 35 °C, and a flow rate of 1.0 mL/min. The eluent used was tetrahydrofuran (THF) containing 2.0% *v*/*v* triethylamine and 0.05% *w*/*v* BHT inhibitor. The sampled amount was 100 μL, and the sample concentration was 3 mg/mL prepared by stirring overnight, followed by filtering through a PTFE syringe filter with a pore size of 0.45 μm. The refractive index detector (RID) detector used was operated at 35 °C. The GPC was calibrated using twelve EasiVial PS-H (2 mL) standards, with molecular weights equal to 162, 580, 1210, 4880, 10,330, 22,790, 75,050, 194,500, 479,200, 885,000, 3,152,000, and 6,570,000 g/mol. The average molecular weight (Mn) was calculated using the Agilent GPC/SEC software 1.3.

#### 2.2.4. Mechanical Properties

Unconfined compression tests on the fluid-filled scaffolds were conducted at each time point using a Lloyd LS5 testing machine with a 5 KN load cell at room temperature. The scaffolds were pre-loaded to 0.5 N and strained to 10% at 0.05 mm min^−1^. The ramp or compression modulus was calculated from the linear portion of the stress-strain curve. The compression was held at 10% strain for 45 min to allow the relaxation phase. This phase allows the fluid to displace from the pores, and it is held till the force reaches an equilibrium value. The equilibrium modulus was calculated as the equilibrium force divided by the cross-sectional area of the scaffold. A dynamic compressive phase at 1% strain at 1 Hz frequency for 10 cycles was then applied after the relaxation phase to calculate the dynamic modulus, which was the average force over the cycles by the cross-sectional area of the scaffold, divided by the strain amplitude. There were four replicates per group. 

### 2.3. Cellular Response to the Degradation of Scaffolds

#### 2.3.1. Human Bone Marrow-Derived Mesenchymal Stem Cell (HBMSC) Culture

Previously harvested HBMSCs [27] were utilised for this study. The cells were harvested from the vertebral body of donors undergoing elective spinal surgery following fully informed consent. Ethical approval for cell collection was provided by the Office for Research Ethics Committees Northern Ireland (ORECNI ref: 18/NI/0174), and approval for continued use was given by the University’s Faculty Research Ethics Committee. Cells were expanded from frozen stocks of the same donor in DMEM low glucose (Sigma Aldrich, Poole, Dorset, UK) supplemented with 10% fetal bovine serum, 2 mM GlutaMAX™-I, and 5 μg/mL Gentamicin (all sources from Sigma Aldrich, Poole, Dorset, UK) and subcultured using 0.25% Trypsin-EDTA and a 1:3 splitting ratio. HBMSCs in passage 4 were used in the experiments.

#### 2.3.2. HBMSC Seeding onto Scaffolds

The scaffolds (n = 3) were sterilised in 70% ethanol for 30 min, rinsed thrice with PBS to ensure excess ethanol was removed, and left to dry overnight in a class II biosafety cabinet. The scaffolds were placed in 24-well plates and incubated in PBS to wet the surface of the scaffolds before cell seeding. Following removal of the PBS, 1 × 10^5^ HBMSCs in 75 µL of culture media were carefully pipetted on top of the scaffolds and incubated for 3 h (37 °C, 5% CO_2_). After the initial cell seeding, 1 mL of cell culture media was added to each well (1 scaffold per well). Media was replaced every alternate day, and the time points were 24 h, 7, 14, 21, and 28 days with three replicates per time point.

#### 2.3.3. Cell Viability on 3D Printed Scaffolds

Cell viability was observed using a live/dead double staining assay (R37601, LIVE/DEAD^®^ Cell Imaging Kit, Thermo Fisher Scientific, Waltham, MA, USA) following the manufacturer’s instructions. Briefly, the scaffolds were washed twice with PBS and then fully submerged in the assay dye. After incubating in the dark for 15 min at room temperature, the scaffolds were sectioned with a sterile scalpel to expose the cross-sectional area which was viewed under a Leica SP8 Multiphoton Confocal microscope. Images were taken from the middle of the scaffold. Image J v1.51q (National Institute of Mental Health, Bethesda, MD, USA) was utilised to calculate the percentage of cell viability in the scaffolds as the percentage of living cells among the total number of cells visualised.

#### 2.3.4. Cell Attachment, Morphology, and Distribution Using SEM

Scaffolds were washed with PBS three times. The cells were then fixed using 2.5% glutaraldehyde in 0.05 M sodium cacodylate buffer for 1 h at 4 °C. Samples were washed with 0.1 M sodium cacodylate buffer once and dehydrated through increasing alcohol gradient from 30% to 100%. Samples were then dried in 1 mL of hexamethyldisilazane (HMDS) overnight at room temperature. Scaffolds were mounted on stubs using double-sided carbon tape and gold coated using an Auto Sputter Coater (Agar Scientific, Stansted, UK), as before. A JEOL 6500 FEG SEM (Advanced Microbeam Inc., Vienna Center, OH, USA) was used to image scaffolds with an accelerating voltage of 5 kV.

### 2.4. Accelerated Degradation

The scaffolds were degraded using an accelerated degradation methodology at an elevated temperature of 47 °C following recommendations from Geddes et al. [9] to investigate the long-term degradation behaviour. The scaffolds (n = 4) were submerged in 10 mL of PBS solution and evaluated at each time point (3, 7, 10, 14, 21, and 28 days) and then characterised by the physiochemical and mechanical properties as detailed above.

## 3. Results

### 3.1. Characterisation of the 3D-Printed Scaffold

**Figure 1:**
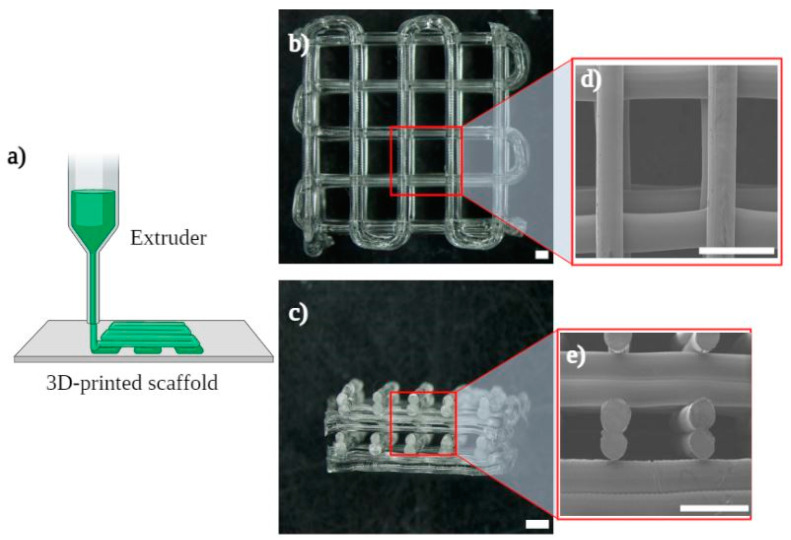
(**a**) Schematic diagram of the 3D printing fabrication process, (**b**) optical microscope image of the fabricated scaffold, (**c**) optical microscope image of the cross-section of the scaffold, (**d**) SEM image of the scaffold, and (**e**) SEM image of the cross-section of the scaffold. Scale bar—1000 µm.

The scaffolds produced using 3D printing (Figure 1a) displayed clear pores with minimal filament defects and an average pore size of 1200 ± 100 µm with a strut size of 330 ± 15 µm (Figure 1b,c). The interconnected porosity of the scaffolds was further established by SEM (Figure 1d,e). The minimal overhangs in between the layers observed in the cross-section of the scaffold confirmed optimum printing conditions. The porosity was 85.9% ± 1.5%. The ramp, equilibrium, and dynamic moduli were 1.65 ± 0.09 MPa, 2.08 ± 0.4 MPa, and 8.55 ± 0.05 MPa, respectively, giving porosity and compressive properties comparable to the properties of native cartilage (ramp modulus 0.45–0.8 MPa, equilibrium modulus 0.5–0.9 MPa, dynamic modulus 5–40 MPa, porosity ~90%) [12].

### 3.2. In Vitro Degradation at Physiological Temperature

#### 3.2.1. Visual Inspection

**Figure 2:**
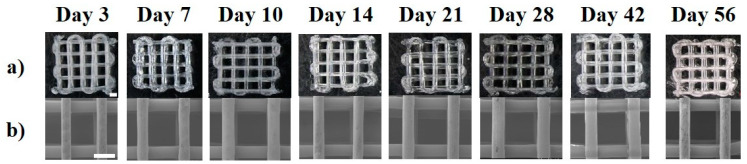
Scaffold appearance at physiological degradation observed under (**a**) optical microscope and (**b**) SEM, scale bar—1000 µm.

The scaffolds did not undergo any notable changes in their visual appearance and structure throughout the degradation study from day 3 to day 56, as shown in Figure 2a, and retained their volumetric structure (Figure 2b). The overall size of the scaffold did not undergo any “visible” swelling; however, on measuring the strand thickness of the scaffolds from the SEM images, a total increase in the strand thickness of 41.8 ± 3.8% to a final strut size of 468 ± 10 µm was recorded at the end of 56 days. The increase in strand thickness appeared gradual across all the time points (Supplementary Table S1). 

#### 3.2.2. Swelling, Change in Mass and pH with Degradation

**Figure 3:**
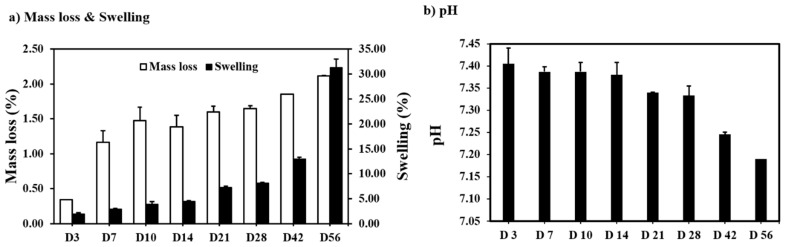
Effect of degradation at physiological temperatures on (**a**) mass loss and swelling (**b**) pH (n = 4, Ave ± std. dev.).

It was observed that when the scaffolds were immersed in PBS, the strand thickness increased from the earliest time point (Figure 3a). This gave a measure of the swelling in scaffolds at physiological temperatures, which were observed to have a maximum swelling of 31.35 ± 1.6% occurring at day 56. The swelling was followed by a reduction in mass; however, this occurred at a slower rate (Figure 3a). A maximum mass loss of 2.12 ± 0.1% was observed on day 56 of physiological degradation. The PBS solution was monitored for changes in pH. A slow decrease in the pH of the PBS buffer from 7.4 ± 0.04 to 7.19 ± 0.04 was observed in 56 days (Figure 3b), indicating the polymer had not degraded enough to begin noticeably releasing oligomers of an acidic nature into the PBS medium [9].

#### 3.2.3. Change in Thermal Properties with Degradation Time

**Figure 4:**
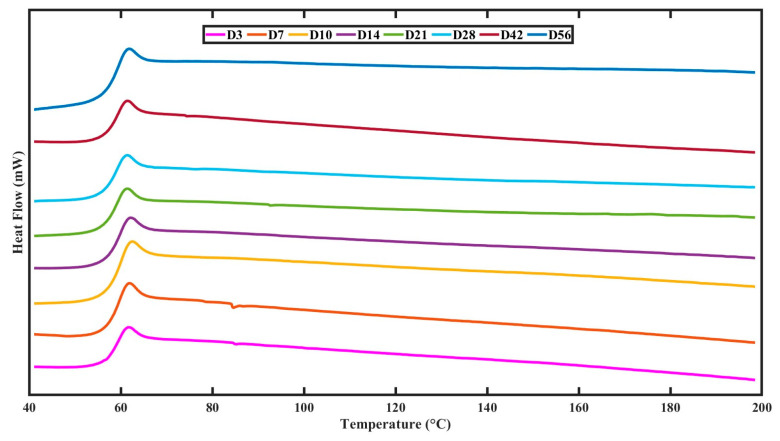
DSC thermograms on the effect of physiological degradation on the glass transition temperature.

The scaffolds displayed a gradual decline in T_g_ of 0.55 ± 0.4 °C (Table 1) from 58.4 ± 0.04 °C to 57.85 ± 0.31 °C for 56 days from the DSC thermograms (Figure 4). The decline in T_g_ observed similar trends to the changes in mass loss, swelling, and pH. There was no crystallisation occurring in these scaffolds for 56 days of degradation at 37 °C (Table 1). 

#### 3.2.4. Change in Molecular Weight with Degradation (GPC)

Mn values of the degrading scaffolds at physiological temperature are presented in Table 2. The Mn of the fabricated scaffold was 125,481. Following in vitro degradation, the Mn had decreased by 39.51% to 75,900 on day 56. The Mw and peak molecular weight (M_p_) decreased by 13.3% and 25.3%, respectively, from day 3 (M_w_ = 212,228, M_p_ = 184,010) to day 56 (M_w_ = 181,049, M_p_ = 135,196) (Supplementary Table S2). 

#### 3.2.5. Change in Mechanical Properties with Degradation

**Figure 5:**
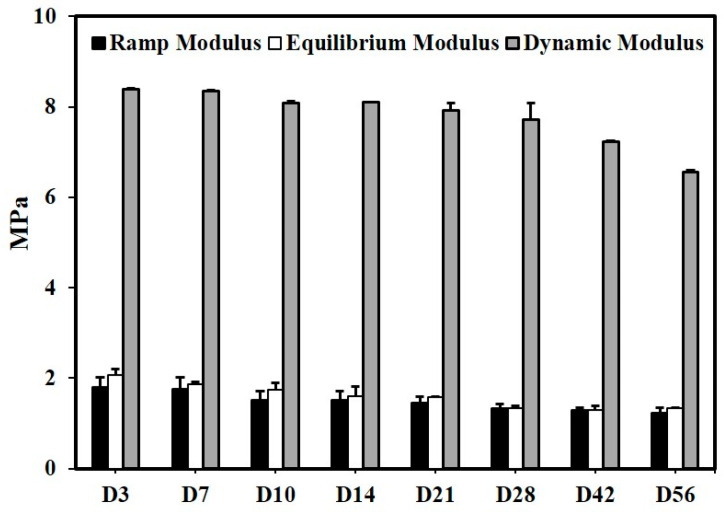
Effect of physiological degradation on compressive properties (n = 4, Ave ± std. dev.).

The compressive properties of the scaffolds underwent gradual changes, with the ramp, equilibrium, and dynamic moduli reducing by 25.90 ± 0.5%, 36.05 ± 0.3%, and 23.27 ± 0.2% to a final of 1.22 ± 0.13 MPa, 1.29 ± 0.02 MPa, and 6.56 ± 0.03 Mpa, respectively, after 56 days of degradation. This change in properties was indicative of the retention of the compressive properties by the scaffolds (Figure 5).

### 3.3. In Vitro Cell Culture Study

#### 3.3.1. Cell Viability Study

**Figure 6:**
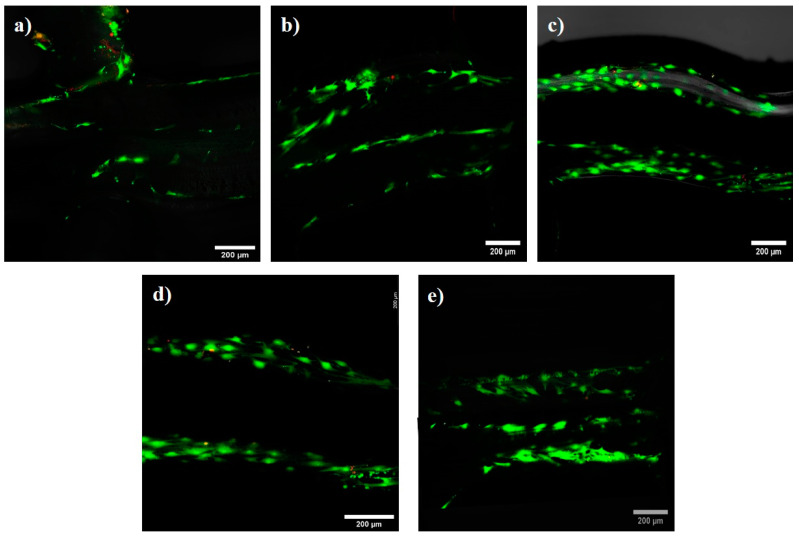
Effect of physiological degradation time on HBMSC viability at (**a**) 24 h, (**b**) 7 days, (**c**) 14 days, (**d**) 21 days, and (**e**) 28 days. Green signal is live cells; red signal indicates dead cells.

**Figure 7:**
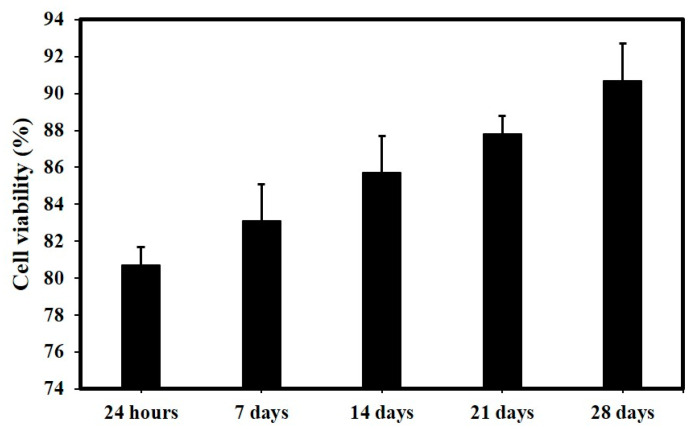
Semi-quantitative measure of Live/dead staining of HBMSCs up to 28 days of cell culture (n = 3, Ave ± std. dev.).

The results of the live/dead cell assay performed on the HBMSC cultured on the scaffolds demonstrated good cell attachment and proliferation during the whole 28-day cell culture period (Figure 6). The interconnected porous nature of the scaffold allowed the HBMSCs cultivated on its surface to enter the three-dimensional structure of the scaffold, and cells were observed on the interior surfaces. The cells also appeared to proliferate with time, with an increase in fluorescent signal observed in the images. A low volume of dead cells (red fluorescence) was observed at all time points. According to the percentage cell viability data shown in Figure 7, about 80.7 ± 2% of live cells were present after 24 h of culturing, which increased steadily to over 90.2 ± 1% remaining viable at 28 days of culture. This result demonstrated that the scaffolds had good cytocompatibility, and the stable degradation of the scaffolds did not prevent cell attachment or proliferation.

#### 3.3.2. Characterisation of Cell Attachment and Morphology Using SEM

**Figure 8:**
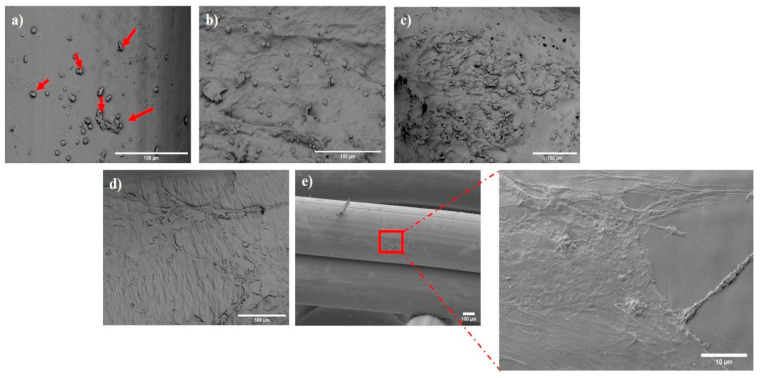
Effect of physiological degradation on cell attachment and morphology at (**a**) 24 h, red arrwows indicating adhered cells on the surface (**b**) 7 days, (**c**) 14 days, (**d**) 21 days, and (**e**) 28 days.

**Figure 9:**
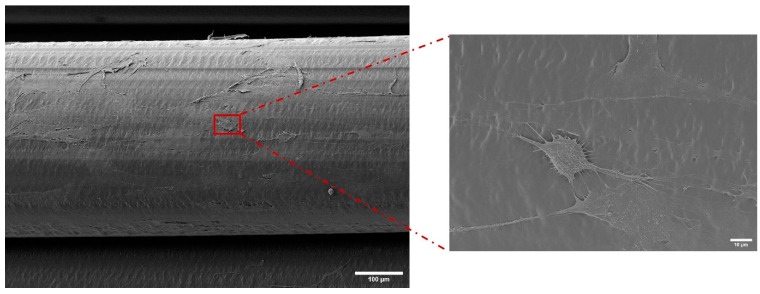
Cell filopodia observed over the PLLGA scaffold struts with cell morphology.

Through SEM imaging, closer observations of cell morphologies were possible. After 24 h of culture, cells adhered to the surface as shown in Figure 8a and were found to proliferate and spread over the surface of the scaffold until day 14. Following this, the cells began to merge and align with the scaffold’s orientation and were dispersed throughout the pores. The majority of the cells had taken on an ellipse- or spindle-shaped appearance and were vertically aligned along the scaffold’s struts by the end of day 28, with cell filopodia observed across the struts (Figure 9). On the surface of the scaffolds, it was discovered that cells had fused and created a full cellular layer. Compared to 24 h of growth, more cells were attached to the scaffold at 28 days (Figure 8e), demonstrating attachment and proliferation of the cells on the degrading scaffold. 

### 3.4. Accelerated Degradation

#### 3.4.1. Visual Inspection

**Figure 10:**
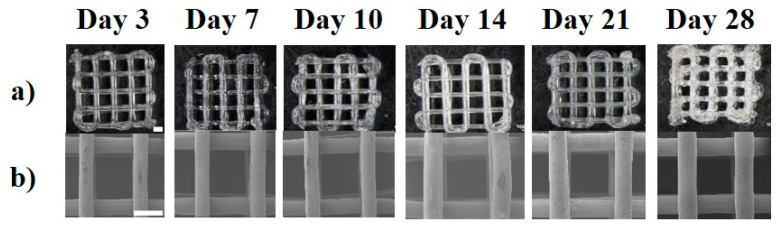
Scaffold appearance at accelerated degradation observed under (**a**) optical microscope and (**b**) SEM, scale bar—1000 µm.

In accelerated degradation conditions, the scaffolds retained their structure for 28 days (Figure 10), with some visible swelling and a whitish appearance in the scaffolds on day 28. A 62.7 ± 3% increase in the strand thickness to a final strut size of 537 ± 15 µm at 28 days was observed (Supplementary Table S3). The gross morphology of the scaffolds showed they retained their structure and were still easily handled during the experiments. 

#### 3.4.2. Swelling, Change in Mass and pH with Degradation

**Figure 11:**
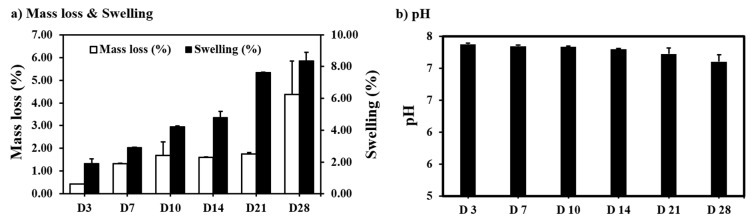
Effect of accelerated degradation on (**a**) mass loss and swelling (**b**) pH (n = 4, Ave ± std. dev.).

An average swelling of 8.37 ± 0.5% after 28 days was recorded, with an average mass loss of 4.38 ± 1.4% (Figure 11a). At accelerated degradation conditions, the pH decreased gradually to 7.11 ± 0.1 at 28 days (Figure 11b). The scaffold retained its bulk properties for 28 days at accelerated degradation.

#### 3.4.3. Change in Thermal Properties with Degradation Time

**Figure 12:**
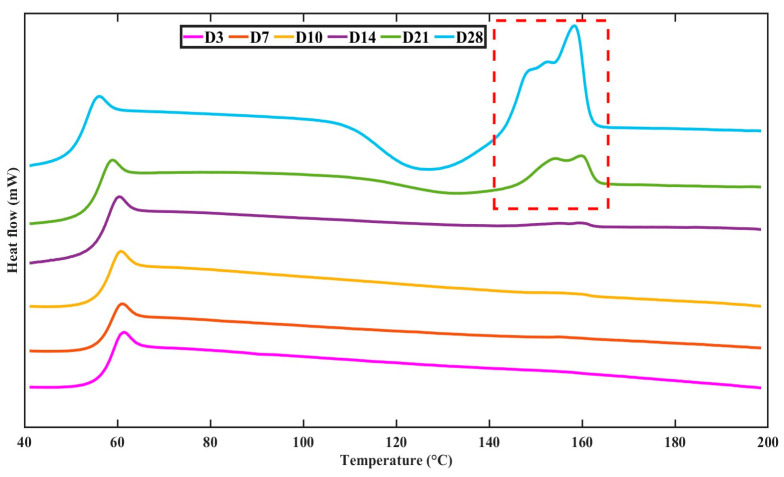
DSC thermograms on the effect of accelerated degradation on the glass transition temperature.

Accelerated degradation conditions induced a more rapid decrease in T_g_ to 53.34 ± 0.03 °C at day 28 with an 11.75 ± 2.3% increase in crystallisation (Table 1). A general trend of decreasing T_g_ and T_m_ with an increase in crystallinity was observed, as marked by the red dashed line in the DSC thermograms in Figure 12. Crystallisation was noted to start from day 21 at 2.97 ± 1% with a T_m_ of 162.17 ± 0.4 °C, followed by an increase in crystallisation to 11.75 ± 1.5% on day 28 with a decrease in the T_m_ to 159.22 ± 0.5 °C.

#### 3.4.4. Change in Molecular Weight with Degradation (GPC)

The molecular weight of the polymer decreased rapidly with time from day 3 to day 28 of in vitro accelerated degradation (Table 3). After in vitro degradation for 28 days, the Mn had decreased by 92.36% to 9585. The M_w_ and peak molecular weight (M_p_) decreased by 88.3% and 84.6%, respectively, from day 3 (M_w_ = 176,009, M_p_ = 137,686) to day 28 (M_w_ = 20,572, M_p_ = 21,201) (Supplementary Table S4). The molecular weight changes were characterised by a rapid decrease from Day 21 to Day 28 at accelerated degradation conditions.

#### 3.4.5. Change in Mechanical Properties with Degradation

**Figure 13:**
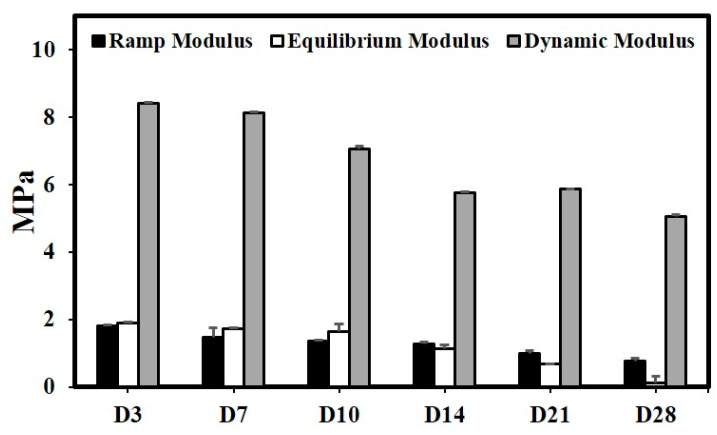
Effect of accelerated degradation on compressive properties (n = 4, Ave ± std. dev.).

The reduction in compressive properties in the scaffolds followed similar trends to the changes in other properties with a gradual reduction up to day 28 by 52.4 ± 1%, 93.4 ± 1%, and 40.9 ± 0.5% to a final of 0.785 ± 0.084 MPa, 0.137 ± 0.180 MPa, and 5.047 ± 0.078 MPa in the ramp modulus, equilibrium modulus, and dynamic modulus properties respectively (Figure 13). The trend observed in both physiological and accelerated degradation conditions was consistent across all its properties.

## 4. Discussion

The overall aim of this study was to engineer a porous scaffold with comparable properties to that of native cartilage and understand the effects of degradation on the physiochemical and mechanical properties of cartilage tissue engineering. First, a porous scaffold (porosity ~86%) was fabricated by 3D printing. The utilisation of 3D printing to fabricate scaffolds has the benefit of enabling defined architectural structures, including specialised models for cartilage tissue engineering. Pore interconnectivity has been shown to have a favourable impact on cell proliferation, migration, and infiltration depth, and a high degree of porosity (~90%), with an open pore network, is best for interacting and integrating with the host tissue [28,29]. The scaffold displayed open pores and an interconnected polymer network with minimal defects in the polymer matrices (Figure 1c,e), with the ramp, equilibrium, and dynamic moduli of the scaffold being comparable with the native cartilage suggesting its suitability to be utilised for cartilage tissue engineering [30,31,32,33].

To determine if a bioresorbable scaffold can provide adequate supporting functions within the timeframe necessary for tissue regeneration, monitoring the changes to the scaffold’s overall characteristics over the degradation phase is crucial. The media chosen to perform the degradation studies was PBS. Although this solution does not replicate body fluid (the absence of enzymes that would be present in the host), studies in the past comparing the degradation profiles of polymers in PBS and in vivo found a close match [34,35,36].

On degrading the scaffolds at physiologically relevant conditions (37 °C), the scaffolds maintained their volumetric structure. This may be because a high porosity results in a low degradation rate in PLGA 85:15 [37], as the polyesters have an acid-catalysed process where the rate of degradation is dependent on the internal concentration of the acidic byproducts. In this study, a high diffusion rate from the porous scaffold may have resulted in a low buildup of acidic products, leading to a gradual degradation of the underlying scaffold.

The degradation traits are in line with the heterogeneous degradation mechanism suggested by Grizzi et al. [38]. On day 56 of degradation, the mass loss reported was within 10% for all the samples tested, which indicated that the scaffolds were still far from total breakdown and retained their original structure. Geddes et al. [9] reported swelling of the PLLGA 85:15 discs began only after a loss in mass; however, in this study, swelling occurred before any mass loss, which could be attributed to increased scaffold wetting due to high porosity. This may have resulted in the polyester linkages within the polymer chains being more vulnerable to hydrolysis by concentrating more water molecules close to them [19]. Since the flow of media in a scaffold is crucial for nutritional and metabolic exchanges in the articular cartilage, the capacity of a scaffold to absorb and retain water with minimal structural changes is a crucial property for creating a viable scaffold for cartilage tissue regeneration [28].

To further confirm the slow release of the acidic oligomers from the polymeric matrix, pH variation during the incubation time was evaluated, as local changes in acidity can influence the growth of tissues in vivo [3]. A small drop in pH over the 56 days confirmed the stable crystalline nature of the polymer in combination with the highly porous structure, aiding in a low build-up of acidic byproducts. The change in thermal characteristics from the DSC thermograms displayed a small decrease in T_g_ of 0.55 °C (Table 2). This is also indicative of a low change in acidity in the PBS solution. A small drop in T_g_ is indicative of surface degradation occurring due to the chain scission of oligomers on the surface of the polymer [39]. PLA has a methyl side chain, which is denser and less hydrophilic than PGA. As a result, lactide-rich PLLGA 85:15 copolymers absorb less water and disintegrate more slowly [40]. It has been stated that the chemical composition, hydrophobicity of the polymer, polymeric device’s architecture, and how easily the scaffold may be accessed by degrading media are significant elements that affect degradation [41].

The changes in molecular weight of the scaffolds provided further information on the degradation mechanism. The molecular weight decreased before any changes in the mass loss. When water diffuses into a polymer, hydrolysis starts, and degradation then happens in two phases where a decrease in molecular weight is seen before any mass loss [8,42]. Furthermore, the gradual decrease in molecular weight of the scaffold to day 56 has been previously observed by Geddes et al. [9]. The low decrease in the molecular weight of the polymer can be associated with random chain scission occurring in the polymer. As the molecular weight in the scaffold drops due to random chain scission, there is initially no mass loss since the chains created are too big to diffuse outside of the polymer matrix. It can be stated that the polymer chains did not reach a critical length corresponding to a significant drop in molecular weight, which resulted in low changes in mass loss and swelling within the fabricated scaffolds [43].

The ramp modulus of the scaffold decreased by 25.90% for over 56 days. The gradual decrease can be attributed to the slow degradation of the scaffold material as ramp testing involves a biphasic response of the scaffold from both fluid pressurisation and the scaffold’s solid phase being deformed [44]. The equilibrium modulus is a measure of the solid phase of the scaffold bearing the compressive load when fluid is no longer flowing through the scaffold. In this regard, the material’s behaviour is comparable to the solid phase of the cartilage [45]. The degradation of the scaffold yielded a maximum decrease in the equilibrium modulus because of the degradation in the solid matrix of the scaffold. Another notable factor is the swelling of the scaffold strands. The fluid retained within the strands of the scaffold provides additional biphasic response even when the interstitial fluid has stopped flowing. The swelling observed over 56 days of degradation, along with mass loss, was comparable to the percentage decrease in the equilibrium modulus, which was related to the decrease in the equilibrium modulus as the scaffold degrades. The decrease in the equilibrium modulus was well within a comparable range of the native cartilage, which showcased the stable functional strength of the scaffold [12].

This phase was followed by a cyclic displacement during a dynamic test. Fluid pressurisation in the pores of the scaffold supports a sizeable portion of the force used in such dynamic testing for normal articular cartilage [44]. Therefore, the dynamic modulus sheds light on both the permeability and compressive stiffness of the designed scaffold during cyclic loading. Furthermore, the dynamic properties of the scaffold are dependent on the amount of strain applied to the scaffold. The articular cartilage experiences a cyclic loading between 10–20% strain amplitude at 1 Hz frequency, depending on the activity [46]. The strain amplitude applied in this case was between 10 and 11%, which could prevent the fluid from escaping, thus resulting in the fluid within the pores of the scaffold bearing the applied compressive strain than the solid matrix. The changes in the dynamic modulus scaffold with degradation are the least in comparison to the other properties (ramp, equilibrium moduli), which could be due to the limited permeability of the fluid from the scaffold at the applied strain rates (1%), which resulted in high levels of interstitial fluid pressurisation from the large pore sizes. Having comparable mechanical properties of the scaffold retained for over 56 days of degradation can have favourable outcomes in vivo as it is within the clinical timeline for cartilage to regenerate [6,7].

The degradation of the scaffolds must not cause any excessive inflammatory response that may affect the regenerating tissue [47]. Studies in the past have found other grades of PLGA (PLGA 50:50, PLGA 75:25) to affect cell viability in vivo within 28 days of incubation due to the significant release of acidic oligomers [3,48]. Hence, a study was conducted to assess the effect of the degrading PLLGA 85:15 scaffolds on HBMSCs. HBMSCs were prominent in all samples, demonstrating successful attachment and biocompatibility of the degrading structure. Visually comparing day 1 images to day 28, an increase in cells is noted, suggesting good attachment, cell proliferation, and a low rate of cell death- all indices of biocompatibility. The cells also aligned along the cell structs, which is a common behaviour for these cells to show spatial alignment [48,49]. The increase in acidic byproducts with degrading PLGA has been known to be a leading cause of cell death [48], but a decrease in pH until 6.8 does not affect cell viability and morphology in vivo [27,48]. In the scaffolds fabricated in this study, the pH after 28 days of degradation had dropped to 7.33 ± 0.02 (Figure 12), which is indicative of the low release of acidic oligomers from the fabricated scaffolds.

The initial molecular weight of the polymer is also known to have a significant effect on cell viability. Sung et al. [3] found that the Mw of PLGA 50:50 scaffolds decreased by 56% in 28 days, which decreased the cell viability to 74% in vitro. The Mw of the scaffold fabricated in this study decreased by 13.3% in 56 days, further demonstrating the stable degradation of the scaffolds. This relative stability in Mw and pH, in conjunction with high levels of porosity and well-connected pores, likely contributes to the positive cell response seen in these initial biocompatibility studies. Furthermore, Geddes et al. [49] evaluated the cytotoxic response of pre-degraded PLLGA 85:15 discs to macrophages, displaying a low cytotoxic effect of the material and lack of inflammatory reaction. It will be necessary to fully understand the cell response to these materials in terms of safety and functionality in more extensive in vitro studies, but the initial results are promising. Furthermore, the study has not incorporated a quantitative measurement of the proliferating HBMSCs on the scaffold which has been semi-quantitatively measured from the live/dead images. This is a limitation in this study.

To understand the long-term degradation behaviour of scaffolds for cartilage tissue engineering, an elevated temperature methodology has been adopted that can decrease the period of evaluation while ensuring the results remain relevant. Fundamentally, the degrading mechanism should closely resemble the in vivo system. On accelerating this degradation, the rate of degradation was more rapid due to higher energy available within the molecules of the polymer [9,22]. The accelerated degradation demonstrated the scaffolds maintained their volumetric structure for 28 days with a white-like appearance and visible swelling in the strands by the end of the time point. The timeline of the swelling and the whitish appearance could depend on the material and architecture of the scaffold. Li et al. [42] noted swelling in PLGA 75:25 solid compression molded scaffolds that turned whitish following 9 weeks of degradation at 37 °C. The increased water absorption due to swelling and change in appearance may be due to water molecules penetrating through the surface and causing a hydrolysis reaction, which increases the creation of water-insoluble low molecular weight polymers and oligomers in the outer layers [18]. For the scaffolds fabricated in this study, there is a higher surface area for the water molecules to penetrate the polymeric strands due to the high porosity. The change in mass loss and swelling goes hand in hand with the stable structural features of the scaffold observed during the degradation. The pH of PBS depleted to 7.11 towards the end of 28 days of degradation. This change in pH is crucial to consider since the hydrolysis reaction is catalysed by elevating the acidity of the degradation media [9]. Once the acidity reaches a critical rate, the degradation mechanism includes both bulk and surface degradation, leading to drastic changes in scaffold properties [50]. However, in the fabricated scaffolds, the change in the physiochemical properties remained stable and had not deteriorated drastically, indicating bulk degradation was not being induced. The DSC thermograms indicated an increase in crystallinity on day 21 and day 28 of accelerated degradation. As more water is absorbed into the polymer due to the large pore size within the scaffold, the oligomer chains released become smaller. The shorter and more mobile chains facilitate their rearrangement into a crystalline structure [9], confirmed by the increase in crystallinity. The molecular weight showed a rapid decline until day 28, when a whitish appearance was observed in the scaffold. Despite the decrease in molecular weight, the scaffolds retained their volumetric structure for 28 days, owing to the crystalline nature of the scaffolds. Furthermore, the mechanical properties of the scaffold observed a rapid decline in comparison to physiological degradation. The decrease in the equilibrium modulus was more drastic at accelerated degradation due to greater swelling and loss in mass of the scaffold. It was observed that this architecture of the fabricated 3D-printed PLLGA scaffolds could be treated for up to 28 days in PBS solution at an elevated temperature without critically affecting its properties.

According to Hukins et al. [43], the degradation rates double when the temperature is raised by 10 °C. However, this does not account for the influence of the nature and molecular weight of the polymer and the geometry of the device, which are factors that can influence the degradation of these scaffolds [33,44,45,46]. On comparing the change in properties at physiological and accelerated degradation conditions, the percentage decrease in mass on day 56 at 37 °C was similar to that of day 28 at 47 °C. A similar trend was also observed for the changes in pH and swelling. However, the change in T_g_ displayed a faster decline at accelerated degradation, with the T_g_ at day 7 at accelerated degradation being comparable to a time point between day 28 and day 42 at physiological degradation. On comparing the molecular weight, the M_n_ on day 56 of degradation at 37 °C was comparable to day 7 of accelerated degradation. Furthermore, on comparing the compressive properties, the decrease in ramp, equilibrium, and dynamic moduli was attained between day 10 and day 14 at accelerated degradation compared with 56 days of physiological degradation. This goes on to show that the degradation progresses through complex changes in the molecular and bulk properties of the scaffold due to multiple factors such as polymer composition, molecular weight, morphology, geometry, and the presence of residual monomers that can result in discrepancies in degradation rates [9].

## 5. Conclusions

In this study, we fabricated and assessed the degradation of biomechanically comparable PLLGA 85:15 scaffolds in a simulated physiological degradation for 56 days and performed an in vitro study to assess the effect of degradation on cell viability for 28 days. Furthermore, an accelerated degradation was also incorporated to understand the suitability of the scaffolds for cartilage tissue engineering. The results revealed that PLLGA 85:15 scaffolds exhibited a stable degradation for 56 days in physiological conditions via surface degradation. The scaffold retained its structural and mechanical features over clinically relevant periods required for cartilage to regenerate in vivo. Additionally, the scaffolds supported cell proliferation and cell migration within the pores, which are critical parameters for tissue regeneration in vivo. Accelerated degradation performed on the scaffolds demonstrated a stable degradation in the scaffolds for 28 days without significant changes in the properties. This demonstrates the suitability of the PLLGA 85:15 scaffolds for cartilage tissue engineering. Future research will focus on long-term degradation effects on cell viability, characterising cellular differentiating capabilities on the PLLGA 85:15 scaffolds for cartilage tissue engineering applications in animal models, as well as tailoring a composite with a hydrogel to improve the formation of the extracellular matrix.

## Figures and Tables

**Table 1:** Thermal properties during degradation at 37 °C and 47 °C.

37 °C			
Degradation Time (Days)	T_g_ (°C)	T_m_ (°C)	Crystallinity (%)
3	58.40 ± 0.01	-	-
14	58.31 ± 0.01	-	-
28	58.27 ± 0.07	-	-
56	57.85 ± 0.31	-	-
47 °C			
3	58.40 ± 0.08	-	-
10	57.10 ± 0.23	-	-
21	54.84 ± 0.91	162.17 ± 0.4	2.97 ± 1%
28	53.34 ± 0.03	159.22 ± 0.5	11.75 ± 1.5%

**Table 2:** Molecular weight of scaffolds during degradation at 37 °C.

37 °C	
Degradation Time (Days)	Mn (g/mol)
0	125,481
3	106,339
7	105,628
10	104,000
14	103,759
21	99,708
28	95,975
42	80,263
56	75,900

**Table 3:** Molecular weight of scaffolds during degradation at 47 °C.

47 °C	Degradation Time (Days)	0	3	7	10	14	21	28
	Mn (g/mol)	125,481	92,506	76,770	64,700	53,481	23,584	9585

## Data Availability

Data is available from the corresponding author on request.

## Supplementary Materials

The following supporting information can be downloaded at: https://www.mdpi.com/article/10.3390/polym15183714/s1, Table S1: Strand thickness changes with degradation at 37 °C; Table S2: Molecular weight of scaffolds during degradation at 37 °C; Table S3: Strand thickness changes with degradation at 47 °C; Table S4: Molecular weight of scaffolds during degradation at 47 °C.
