# Supplementary material for: In Vitro Degradation of 3D-Printed Poly(L-lactide-Co-Glycolic Acid) Scaffolds for Tissue Engineering Applications
Source: Polymers (Basel). 2023 Sep 9;15(18):3714. doi: 10.3390/polym15183714 (PMC10534938; doi:10.3390/polym15183714)
Supplement: Supplementary file 1 [file polymers-15-03714-s001.zip › polymers-2561415-supplementary.pdf]

polymers

MDPI

Supplementary Materials

In Vitro Degradation of 3D-Printed Poly(L-lactide-Co-Glycolic Acid) Scaffolds for Tissue Engineering Applications

Anushree Ghosh Dastidar [1], Susan A. Clarke [2], Eneko Larrañeta [3], Fraser Buchanan [1] and Krishna Manda [1,*]

[1] School of Mechanical and Aerospace Engineering, Queen's University Belfast, Belfast BT9 5AH, UK; aghoshdastidar01@qub.ac.uk (A.G.D.); f.buchanan@qub.ac.uk (F.B.)
[2] School of Nursing and Midwifery, Queen's University Belfast, Belfast BT9 7BL, UK; s.a.clarke@qub.ac.uk
[3] School of Pharmacy, Queen's University Belfast, Belfast BT9 7BL, UK; e.larraneta@qub.ac.uk
* Correspondence: k.manda@qub.ac.uk

Table S1. Strand thickness changes with degradation at 37°C.

37 °C	
Degradation time (days)	***Strand thickness (µm)***
0	330±15
3	413±10
7	429±12
10	432±11
14	449±09
21	456±13
28	452±15
42	465±14
56	468±10

Table S2. Molecular weight of scaffolds during degradation at 37°C.

37 °C		
Degradation time (days)	***Mw (g/mol)***	***Mp (g/mol)***
0	214,335	195,023
3	212,228	184,010
7	181,969	147,769
10	175,086	134,965
14	195,414	163,756
21	184,010	135,196
28	193,324	152.226
42	170,148	133,968
56	181,049	135,196

Table S3. Strand thickness changes with degradation at 47°C.

**47 °C**	
Degradation time (days)	***Strand thickness (µm)***
0	330±15
3	422±09
7	423±10
10	436±11
14	460±12
21	462±13
28	537±15

Table S4. Molecular weight of scaffolds during degradation at 47°C.

**47 °C**		
Degradation time (days)	***Mw (g/mol)***	***Mp (g/mol)***
0	214,335	195,023
3	176,009	137,686
7	140,321	114,715
10	120,653	98,546
14	93,895	74,023
21	41,665	43,812
28	20,752	21,201
